# Tissue- and Cell-Specific Mitochondrial Defect in Parkin-Deficient Mice

**DOI:** 10.1371/journal.pone.0099898

**Published:** 2014-06-24

**Authors:** Maria Damiano, Clément A. Gautier, Anne-Laure Bulteau, Rosa Ferrando-Miguel, Caroline Gouarne, Marc Giraudon Paoli, Rebecca Pruss, Françoise Auchère, Caroline L'Hermitte-Stead, Frédéric Bouillaud, Alexis Brice, Olga Corti, Anne Lombès

**Affiliations:** 1 Inserm, U 975, CRICM, Hôpital de la Pitié-Salpêtrière, Paris, France; 2 UPMC Université Paris 06, UMR_S975, Paris, France; 3 CNRS, UMR 7225, Paris, France; 4 Inserm U 1016, Institut Cochin, Paris, France; 5 CNRS UMR 8104, Paris, France; 6 Université Paris 05, UMR_S1016, Paris, France; 7 Trophos, SA Parc Scientifique de Luminy Case, Marseille, France; 8 Laboratoire Mitochondries, Métaux et Stress Oxydatif, Département de Pathologie Moléculaire et Cellulaire, Institut Jacques Monod, Université Paris-Diderot/CNRS, Paris, France; 9 AP-HP, Hôpital de la Salpêtrière, Department of Genetics and Cytogenetics, Paris, France; Instituto de Investigación Hospital 12 de Octubre, Spain

## Abstract

Loss of Parkin, encoded by *PARK2* gene, is a major cause of autosomal recessive Parkinson's disease. In Drosophila and mammalian cell models Parkin has been shown in to play a role in various processes essential to maintenance of mitochondrial quality, including mitochondrial dynamics, biogenesis and degradation. However, the relevance of altered mitochondrial quality control mechanisms to neuronal survival *in vivo* is still under debate. We addressed this issue in the brain of *PARK2^−/−^* mice using an integrated mitochondrial evaluation, including analysis of respiration by polarography or by fluorescence, respiratory complexes activity by spectrophotometric assays, mitochondrial membrane potential by rhodamine 123 fluorescence, mitochondrial DNA content by real time PCR, and oxidative stress by total glutathione measurement, proteasome activity, *SOD2* expression and proteins oxidative damage. Respiration rates were lowered in *PARK2* ^−/−^ brain with high resolution but not standard respirometry. This defect was specific to the striatum, where it was prominent in neurons but less severe in astrocytes. It was present in primary embryonic cells and did not worsen *in vivo* from 9 to 24 months of age. It was not associated with any respiratory complex defect, including complex I. Mitochondrial inner membrane potential in *PARK2^−/−^* mice was similar to that of wild-type mice but showed increased sensitivity to uncoupling with ageing in striatum. The presence of oxidative stress was suggested in the striatum by increased mitochondrial glutathione content and oxidative adducts but normal proteasome activity showed efficient compensation. *SOD2* expression was increased only in the striatum of *PARK2^−/−^* mice at 24 months of age. Altogether our results show a tissue-specific mitochondrial defect, present early in life of *PARK2^−/−^* mice, mildly affecting respiration, without prominent impact on mitochondrial membrane potential, whose underlying mechanisms remain to be elucidated, as complex I defect and prominent oxidative damage were ruled out.

## Introduction

Mitochondrial dysfunction has long been thought to play a key role in Parkinson's disease (PD) pathogenesis. Parkinsonian syndromes are induced in humans and animals by complex I inhibitors, such as 1-methyl-4-phenyl-1,2,3,6-tetrahydropyridine (MPTP) or rotenone, and complex I activity has been reported to be reduced in different tissues from patients, suggesting that this defect may contribute to neuronal degeneration in PD [1], [2]. Mitochondrial dysfunction has also been suggested to contribute to oxidative damage and apoptosis in idiopathic PD brains [3]. Identification of genetic causes of familial PD reinforced a central role for mitochondria in PD pathogenesis [4], [5]. Indeed most autosomal recessive forms were found to be caused by the alteration of a gene encoding a protein localized to mitochondria, either in particular contexts, as in the case of Parkin [6] and DJ-1 [7], or constitutively for PINK1 [8]. Furthermore, studies in various models and from many independent laboratories have provided evidence for interaction between the *PARK2* and *PINK1* genes and their protein products in a common pathway centered on maintenance of mitochondrial quality [9]. In cell models Parkin and PINK1 regulate the elimination of dysfunctional mitochondria through mitophagy [10]–[15]. Parkin and PINK1 have also been involved in mitochondrial fission and fusion [16]–[18], mitochondrial transport [19], [20], and mitochondrial biogenesis [21], [22], processes that are also relevant to mitochondrial quality. Consistent with these findings, *PARK2* or *PINK1* inactivation have been reproducibly linked with partial mitochondrial depolarization [23]–[25], reduced respiration rates [22], [24], [26], [27] and/or reduction in the enzymatic activity of complex I of the mitochondrial electron transport chain [24]–[30].

However, the *in vivo* relevance of the PINK1/Parkin-dependent mitochondrial quality control mechanisms for neuronal cells has recently been questioned [31], [32]. In addition, PINK1- or Parkin-deficient mouse models have less consistently shown mitochondrial defects than cellular models, possibly due to differences in the ages and tissues examined and in the methodologies used [26], [33]–[37]. Several groups, including ours, have generated Parkin-deficient mice and shown that, in general, they do not present with overt signs of neuronal degeneration in the nigrostriatal pathway, even at high ages [38]–[42]. In some cases, including our model, moderate alterations in dopamine handling evocative of presymptomatic changes have been reported in the striatum, such as increased extracellular dopamine content, enhanced dopamine metabolism via monoamine oxidase and decreased motor activation following amphetamine-induced dopamine-release [38], [39]. Therefore, despite the lack of manifest parkinsonian phenotype, these mice may be valuable for investigation of early disease-related modifications and compensatory mechanisms. To clarify discrepancies in the literature regarding the role of mitochondrial dysfunction in these models [35], [37], we have investigated mitochondrial functions, including respiration, respiratory complexes activity and inner membrane potential, in mice carrying a germline homozygous deletion of *PARK2* exon 3 [39].

To address potential technical biases underlying the literature discrepancies, we analyzed respiration in different mitochondrial and tissue preparations, including crude mitochondrial pellets from striatum and cortex, purified mitochondria from whole brain, and post-nuclear supernatants from midbrain and striatum; we also examined primary embryonic neurons and astrocytes from striatum or cortex. We provide evidence for tissue- and cell-type-specific reduction of mitochondrial respiratory capacity in *PARK2^−/−^* mice. These defects were observed in the absence of overt oxidative damage, decreased mitochondrial membrane potential or defective enzymatic activities of the respiratory chain complexes, thus leaving open their underlying mechanisms.

## Materials and Methods

### Ethics statement

All experiments were approved by the “Comité d'Ethique pour l'expérimentation animale Charles Darwin” (Ce5/2009/052).

### Generation of experimental groups

*PARK2* ^−/−^ mice were generated and brought into the C56Bl/6j genetic background as previously described [39], [42]. Experimental groups of age-matched littermate *PARK2^−/−^* and wild type mice were generated by intercross of heterozygous *PARK2^−/+^* mice. Genotypes were determined by PCR amplification [42]. After weaning, male and female mice were separated and maintained at a maximum of five littermates per cage, with food and water ad libitum, and at constant temperature (20°C+/−2°C) with a 08:00–18:00 light cycle. The experiments were performed on female mice (n = 42 per genotype) as follows, with n corresponding to the number of animals per genotype, 24-month-old mice were used for respiration analyses on crude mitochondrial pellets (n = 8), purified mitochondrial pellets (n = 2) or post-nuclear supernatants (n = 8), and oxidative stress evaluation with glutathione and proteasome studies (n = 5), 9-month-old mice for respiration analyses on post-nuclear supernatants (n = 7), 12-month-old mice for respiratory chain complexes assays (n = 7), and oxidative stress evaluation with glutathione and proteasome studies (n = 5). For all applications, mice were killed by decapitation, without prior anesthesia.

### Tissue preparation

After mice decapitation, liver and brain were rapidly removed and the regions of interest dissected on ice. All samples were manually homogenized using a Dounce homogenizer with a Teflon pestle. The scheme depicting the different tissue preparations and their use is shown in Figure S1.

Crude mitochondrial pellets from the striatum or cortex were obtained by homogenization in 9 volumes of Buffer A (50 mM Tris HCl, 5 mM Glucose, 1 mM Pyruvate, 1 mM EGTA, 150 mM KCl, pH 7.5). Centrifugation for 5 min at 1000 g gave post-nuclear supernatant. The nuclei and tissue particles in the pellet was subjected to a second identical cycle of homogenization and centrifugation. The two post-nuclear supernatants were collected and centrifuged for 10 min at 10,000 g to obtain the crude mitochondrial pellet that was diluted in 120 and 240 µl of Buffer A for striatal and cortical mitochondria respectively. These suspensions were used for respiration and protein assay. Between 5 and 10 µg mitochondrial proteins were used per run of respiration.

Purified brain mitochondria were obtained by homogenization of a whole brain in 1 mL Mannitol-Sucrose (MS) isolation medium (225 mM mannitol, 75 mM sucrose, 1 mM EGTA, 5 mM HEPES, pH 7.4) containing 0.1 mg/mL fatty acid-free bovine serum albumin (FAF-BSA) [43]. The homogenate volume was brought to 1.5 ml. The supernatant of a first centrifugation during 6 min at 500 g was centrifuged during 10 min at 14 000 g to obtain the crude mitochondrial pellet that was diluted in 200 µl of 12% Percoll solution in MS buffer, layered on top of a discontinuous Percoll gradient (200 µl of 12% Percoll on top of 1 mL of 24% Percoll in MS buffer), and centrifuged during 18 min at 17 000 g. The resulting mitochondrial pellet was washed twice with MS buffer with centrifugation during 7 min at 17 000 g the first time and during 5 min at 14 000 g the second time. The last purified mitochondrial pellet was diluted in 100 µl MS buffer and used for respiration (5 µg of mitochondrial proteins per run) and protein assays.

Liver, ventral midbrain and striatal post-nuclear supernatants were obtained by homogenization in 9 volumes of 300 mM sucrose, 1 mM EGTA, 5 mM Tris HCl pH 7.4, followed by centrifugation during 10 minutes at 1000 g. The post-nuclear supernatants were directly used for respiration and protein assays, as well as for the analysis of inner mitochondrial membrane potential. Each run of respiration assay used 300 µg of protein of post-nuclear supernatant from midbrain or striatum and 1 mg from liver.

Primary striatal or cortical neurons were prepared at embryonic day 16 by a method adapted from Friedman et al [44]. In summary, following centrifugation through a BSA cushion, primary neurons were seeded onto poly-ornithine and laminin coated Seahorse 24-well plates (175,000 cells/well for striatal neurons and 140,000 cells/well for cortical neurons) and cultured for 7 days at 37°C, 5% CO2 in Neurobasal medium supplemented with 2% B27, 1 mM sodium pyruvate and 1 mM glutamax.

Astrocytes were isolated from striatum or cortex of newborn mice as previously described [45] and cultured in poly L-lysine coated tissue culture flasks (75 cm2) for 7 days. Astrocytes were then trypsinized, plated in Seahorse 24-well plates (40,000 cells/well) and cultured for another 4 days at 37°C, 5% CO2 in Dulbecco's modified Eagle's medium F12 supplemented with 10% fetal bovine serum and1% penicillin streptomycin.

### Respiration assays

For mitochondrial preparations or post-nuclear supernatants, the rate of oxygen consumption was measured in a Clark-type electrode. Mitochondrial pellets were analyzed by classical respirometry using Hansatech electrode (Cambridge, UK). The respiration medium was 100 mM KMES, 5 mM KH_2_PO_4_, 1 mM EGTA, 3 mM EDTA, 0.4% w/v FAF-BSA, pH 7.4 for crude mitochondrial pellets. It was 125 mM KCl, 1 mM MgCl_2_, 2 mM KH_2_PO_4_, 20 mM HEPES, 200 µM EGTA, 0.2% w/v FAF-BSA, pH 7.4 for purified mitochondria. Post-nuclear supernatants were analyzed by high resolution respirometry with an Oroboros apparatus (Innsbruck, Austria) using 100 mM KCl, 40 mM sucrose, 10 mM TES, 5 mM MgCl2, 1 mM EGTA, 0.4% w/v FAF-BSA, pH = 7.2 as respiration medium.

Common recorded parameters were the respiration rate in the presence of 10 mM glutamate, 5 mM malate and 1 mM ADP, in the presence of 1 µg/mL oligomycin, a complex V inhibitor, and in the presence of 1 mM KCN ( = non-respiratory oxygen consumption). These parameters allowed the calculation of state 3 respiration = respiration in the presence of substrates+ADP - non-respiratory oxygen consumption, state 4 respiration = respiration in the presence of oligomycin - non-respiratory oxygen consumption, and respiratory control ratio = state 3/state 4, evaluating the efficiency of the oxidative phosphorylation coupling. Two additional parameters were recorded with Oroboros electrode: basal respiration (in the presence of substrates before ADP addition) and maximal respiration rate (maximal rate obtained by progressive additions of 1.25 µM carbonyl cyanide m-chlorophenyl hydrazone (cccp), a protonophore dissipating the inner membrane potential, performed after the step with addition of oligomycin). These additional parameters were used to calculate the respiratory reserve = maximal respiration - basal respiration.

For primary neurons and astrocytes, respiration was analyzed in a XF24 extracellular flux analyzer (Seahorse Bioscience) in the presence of 25 mM glucose [46]. Subsequent steps included complex V inhibition by oligomycin, inner membrane potential dissipation by tri-fluorocarbonylcyanide phenylhydrazone (fccp), and respiration inhibition by rotenone and antimycin, complex I and complex III inhibitors respectively. For each cell type, preliminary experiments determined the concentration of these agents to optimally inhibit or stimulate respiration without inducing toxicity (as mentioned in the figure legend). To measure the relative cell density and viability in each well after respiration measurements, cells were incubated during 20 minutes with 2 µg/ml calcein-AM, a cell-permeant fluorogenic esterase substrate whose fluorescence emission was measured at 532 nm, with excitation set at 650 nm. For astrocytes, calcein global fluorescence was not proportional to cell number seeded, so the non-mitochondrial OCR, which measures non mitochondrial oxidase activity, was used to control for cell density equivalence. Cells from wild type and *PARK2^−/−^* mice were seeded in different wells of the same plate and only experiments with similar cell densities were used for analysis. The recorded parameters were initial respiration, state 4 respiration rate (respiration under oligomycin inhibition minus non-respiratory oxygen consumption under rotenone and antimycin) and maximal respiratory rate (respiration under fccp minus non-respiratory oxygen consumption). The initial respiration rate slightly differs from the basal respiration recorded with mitochondrial pellets or post-nuclear supernatants because ADP level cannot be controlled in intact cells. The difference between maximal and initial respiration was thus named spare respiratory capacity instead of respiratory reserve. Coupling efficiency was evaluated by the ratio between initial respiration rate and respiration under oligomycin (both after subtraction of the non-respiratory oxygen consumption).

### Mitochondrial activities

The maximal velocity of respiratory complexes and citrate synthase activities was evaluated using standardized spectrophotometric assays used in the diagnosis of human mitochondrial diseases [47] while complex V activity was assayed using the ATP hydrolysis assay [48].

### Determination of the mitochondrial transmembrane potential

The mitochondrial membrane potential was evaluated in post-nuclear supernatants using the fluorescent probe rhodamine 123, which accumulates in the mitochondrial compartment according to the Nernst law [49]. Incubation was performed at room temperature with 2 to 5 µg protein of post-nuclear supernatants/mL of respiration medium with 250 nM rhodamine 123, and in diverse bioenergetic conditions: condition A = substrates (10 mM glutamate+5 mM malate), condition B = A+1 mM ADP, condition C = B+1 µM oligomycin, condition D = C+1 µM cccp, condition E = C+2 µM cccp, condition F = C+4 µM cccp, condition G = C+8 µM cccp, condition H = C+10 µM cccp and 1 mM KCN. Reading was performed with an Accuri flow cytometer. Results were expressed in mV as calculated by Nernst equation after normalization of the fluorescence to the size of the objects as measured by their forward scatter, taking into account the proportion of mitochondrial objects (% of energizable objects upon maximal energization), evaluating the mitochondrial volume in the optic chamber of Accuri flow cytometer (estimated for brain at 3% of the volume analyzed), and using condition H for evaluation of the zero fluorescence.

### Quantification of mitochondrial DNA

Quantification of mitochondrial DNA was performed in striatum by quantitative real time PCR using as standard a plasmid with the murine mitochondrial DNA-encoded *MT-CO2* gene as an insert [50]. Results were expressed as copy number of mitochondrial DNA/10 pg of total DNA, which roughly represent a normal diploid genome.

### Determination of cytosolic and mitochondrial glutathione levels

Total glutathione levels were determined using 20 µg proteins of crude mitochondrial pellet or cytosol from striatum and midbrain with a modified version of the recycling enzymatic assay based on the reduction of each GSSG molecule to give two GSH molecules [51]. Standard curves were obtained with various concentrations of GSSG and the values were thus expressed as GSSG equivalent. Total glutathione content therefore equals 0.5 GSH+GSSG and is expressed in nmoles of glutathione/mg of protein.

### 20S proteasome peptidase activity

The chymotrypsin-like activity of the proteasome was assayed in cytosol from striatum and midbrain with the fluorogenic peptide succinyl-Leu-Leu-Val-Tyr-amidomethylcoumarin (LLVY-AMC) as substrate. 20 µg cytosolic proteins were incubated with 12.5 µM substrate in 25 mM Tris, pH 7.5 in a final volume of 200 µl [52]. Enzymatic kinetics was monitored in a temperature-controlled microplate fluorimetric reader (Fluostar Galaxy; BMG, Stuttgart, Germany). Excitation/emission wavelengths were 350 nm/440 nm.

### Western blot analyses

30 µg of proteins of crude mitochondrial pellets from striatum or midbrain were solubilized overnight at room temperature in sample buffer at a SDS/protein ratio = 30. Proteins were separated by electrophoresis in 4–20% pre-cast gradient gels (Biorad, Hertfordshire, UK) and transferred on nitrocellulose membrane (Protran Whatman, Dassel, Germany). Antibodies were rabbit polyclonal anti-SOD2 from Sigma and anti-nitrotyrosine from Molecular Probes, mouse monoclonal anti-VDAC from Mitoscience and rabbit anti-4-hydroxynonenal (HNE) produced in the team [52]; they were visualized using peroxidase-conjugated secondary antibodies from Jackson ImmunoResearch (Newmarket, UK).

### Statistical methods

Statistics were performed with SigmaPlot 12.5 software. Non parametric tests were used unless the data were shown to have a normal distribution allowing the use of parametric tests. The numbers given indicate independent measurements. Animals were not pooled but always individually evaluated. Mean comparisons were performed with Mann and Whitney test or unpaired Student t test while paired comparisons were done by the Wilcoxon signed-rank test or paired Student t test. Multiple comparison with the Holm-Sidak test was used after verification of equal variance to address the relative influence of age, genotype and their interaction.

## Results

### High resolution respirometry and cell-type specific analysis detect a respiration defect in PARK2^−/−^ mice

Reductions in uncoupled respiratory rates have previously been observed in the striatum or the substantia nigra of *PARK2^−/−^* mice by two independent groups, with however partial inconsistency with respect to the associated state 3 and state 4 respiration rates [35], [37]. Both reports relied on the analysis with standard oxygraphy of relatively crude mitochondrial pellets obtained by two successive centrifugation steps. Under these conditions, the observed respiratory control ratios (state 3/state 4 respiration) were low (below 3.5), reflecting relatively poor coupling of the electron transport chain with ATP synthesis [35], [37].

**Figure 1:**
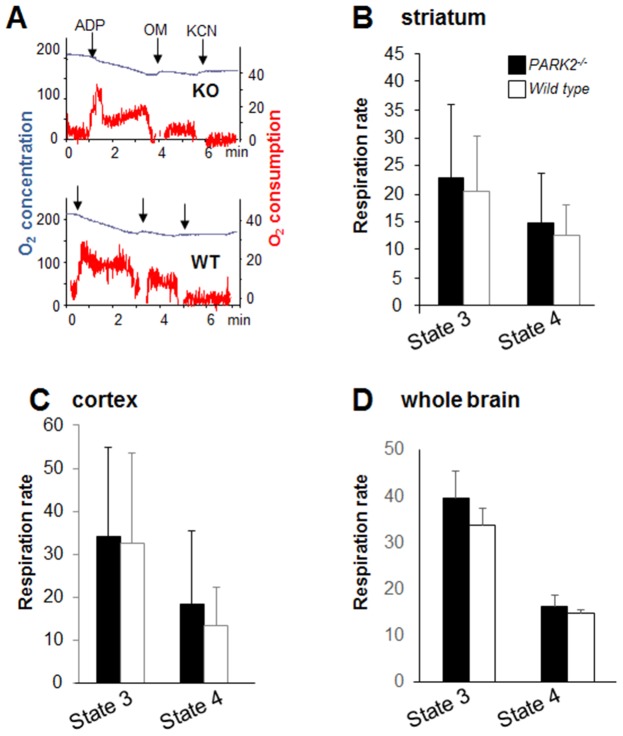
Respiration was similar in brain mitochondrial preparations from 24-month-old *PARK2^−/−^* and wild type mice examined with standard oxygraphy. (**A**) Representative experiment with a crude mitochondrial pellet from striatum. Blue curve = oxygen (O_2_) concentration expressed as nmol/mL; red curve = rate of oxygen consumption expressed as nmoles O_2_/mL and min; ADP = addition of 400 µM ADP; OM = addition of 1 µg/mL oligomycin, a complex V inhibitor; KCN = addition of 1 mM KCN (non-respiratory oxygen consumption); (**B, C, D**) Bars showing state 3 respiration (respiration in the presence of substrates +ADP – non-respiratory oxygen consumption) and state 4 respiration (respiration in the presence of oligomycin – non-respiratory oxygen consumption) on complex I substrates (glutamate+malate); black bars = *PARK2^−/−^*, white bars = wild type; data are expressed as nmoles O_2_/mL and mg proteins and are shown as means ± SD; two animals, one per genotype, were analyzed the same day; the numbers between brackets indicate the number of individual animals of each genotype; (B) results from crude mitochondrial pellets from striatum (n = 6), (C) crude mitochondrial pellets from cortex (n = 7) and (D) purified mitochondria from whole brain (n = 2).

We performed similar experiments in aged mice considering that they were more likely to express a defect because of the lack of neurodegenerative phenotype in *PARK2^−/−^* mice [35], [39], [41] and the relationship of PD with ageing. Crude mitochondrial pellets from striatum and cortex were prepared from the brain of 24-month-old mice (Figure S1). State 3 and state 4 respiration as well as the respiratory control ratio were similar in *PARK2^−/−^* and wild type mice (Figure 1A, B, C). As in the previous studies, the respiratory control ratios were relatively low (Figure S2).

Considering that mild respiration defects might not be detectable in crude mitochondrial pellets we next prepared whole brain purified mitochondria from 24-month-old mice and found similar state 3 and state 4 respiration as well as respiratory control in *PARK2* ^−/−^ and wild-type mice (Figure 1D). Although mitochondrial enrichment of the preparation was shown by the higher specific respiratory rates as compared to those of crude mitochondrial pellets, the respiratory control ratio of these purified mitochondria was only slightly increased (Figure S2). Considering the need to use whole brain instead of specific brain region, the improvement of respiratory control ratio did not appear sufficient to pursue that procedure in a higher number of animals.

**Figure 2:**
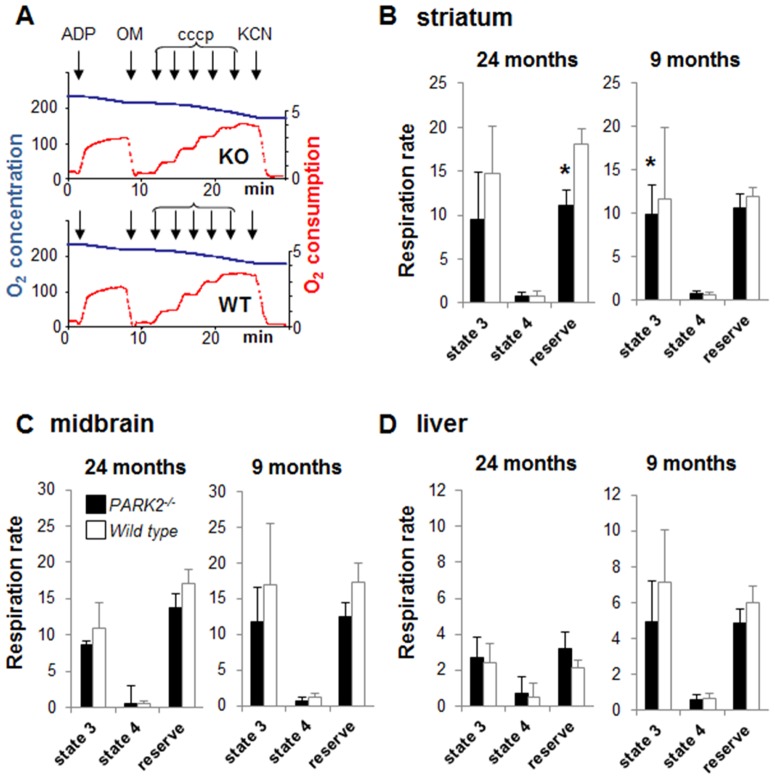
High resolution respirometry reveals a respiration defect in *PARK2^−/−^* mice. (**A**) Representative experiment with striatal post-nuclear supernatant. Blue curve = oxygen concentration (O_2_) expressed as nmol/mL; red curve = rate of oxygen consumption expressed as nmoles O_2_/mL and min; successive additions: ADP = 1 mM ADP in the medium with 10 mM glutamate+5 mM malate; OM = 1 µg/mL oligomycin, cccp = successive additions of 1.25 µM (up to the maximal respiration rate independent from ATP synthase capacity); KCN = 1 mM KCN (non-respiratory oxygen consumption); (**B, C, D**) Bars showing state 3 respiration, state 4 respiration and respiratory reserve on complex I substrates (glutamate+malate) in post-nuclear supernatants from (B) striatum, (C) midbrain and (D) liver of 24- and 9-month-old *PARK2^−/−^* (black bars) and wild type mice (white bars); all data are expressed as nmoles O_2_/mL and mg proteins and shown as means ± SD; two animals, one per genotype, were analyzed the same day; the data were obtained from 6 to 8 individual animals of each genotype. * *p*<0.05 using Mann and Whitney test. In liver, state 3 respiration rate and respiratory reserve significantly decreased with age in both wild type mice (p = 0.011 and <0.001 when comparing state 3 respiration and respiratory reserve respectively between 9 and 24 months of age using Mann and Whitney test) and in *PARK2^−/−^* mice (p = 0.019 and <0.001 when comparing state 3 respiration and respiratory reserve respectively between 9 and 24 months of age using Mann and Whitney test).

Furthermore we reasoned that altered mitochondria were likely to be eliminated during mitochondrial purification. That procedure might thus be inappropriate when addressing mitochondrial quality control as is the case in the absence of Parkin. Very low respiration rates such as those observed without mitochondrial enrichment can only be analyzed with high resolution respirometry using Oroboros technology. We therefore optimized this technique for the analysis of post-nuclear supernatants. The relative rapidity of this tissue preparation allowed the parallel study of striatum, midbrain and liver from each mouse. With this method the respiratory control ratios were above 10 in midbrain and striatum, indicating excellent mitochondrial preservation. These ratios were similar between *PARK2^−/−^* and wild type mice (Figure S2). An example of curve obtained with 300 µg striatum post-nuclear supernatant is shown in Figure 2A. 24-month-old *PARK2^−/−^* mice showed significant decreased respiratory reserve in striatum when compared to wild type mice (p = 0.028 using Mann and Whitney test). The concomitant decrease of state 3 respiration in striatum just failed to reach significance (p = 0.06 with Mann and Whitney test) (Figure 2B). State 4 respiration was not affected. The decreased state 3 respiration and respiratory reserve in midbrain did not reach significance (Figure 2C) and liver respiration was similar in both genotypes (Figure 2D).

Using the same approach we then studied younger mice at 9 months of age to address the presence of the respiratory defect earlier in life. *PARK2^−/−^* mice presented with a decrease in state 3 respiration rate and respiratory reserve in midbrain and striatum that was similar to the pattern observed in aged mice (Figure 2B). As previously observed in aged mice, the decrease was only significant in the striatum (p = 0.05 for state 3 respiration with Mann and Whitney test).

Liver showed a significant age-associated decrease in respiratory reserve capacity in both genotypes (Figure 2D). In contrast the respiratory capacity in striatum and midbrain post-nuclear supernatants was not modified by age.

**Figure 3:**
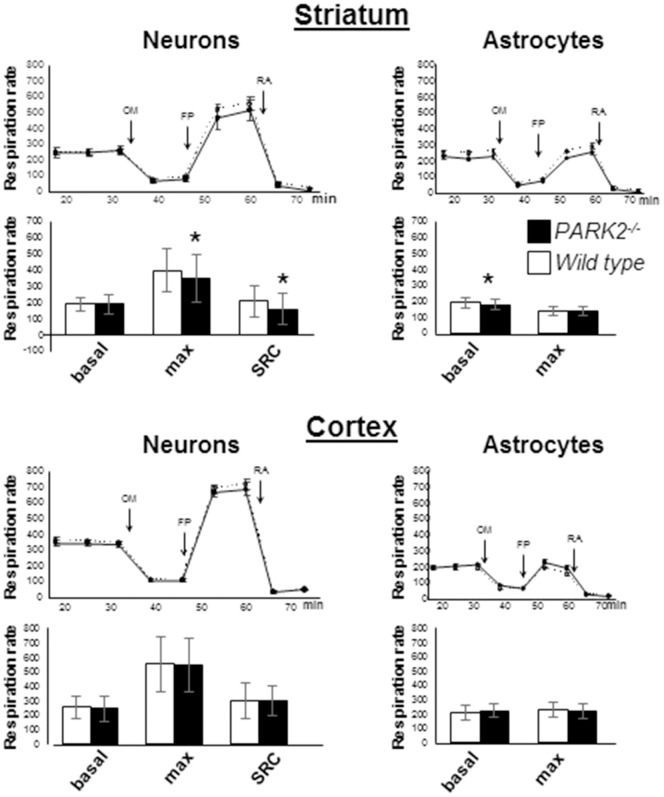
Maximal respiration is reduced in striatal neurons from *PARK2^−/−^* mice. Representative experiments with neurons or astrocytes from striatum or cortex: the traces represent the evolution of the respiration rate (in pmol O_2_/minute) in Seahorse plates seeded with cells from *PARK2^−/−^* (black circles) and wild type (white circles) mice. Sequential additions are: oligomycin (OM) (0.25 µg/ml for neurons and 0.5 µg/ml for astrocytes), fccp (FP) (3 µM for cortical neurons and 1 µM for other cells), and rotenone+antimycine (RA) (50 nM rotenone+150 µg/ml antimycin A). Bars below the traces show the means and SD of basal respiration, maximal respiration and spare respiratory capacity (SRC = maximal – basal respiration) of 3 independent tests, each performed in 6 to 12 independent wells. * *p*<0.05 using Mann and Whitney test.

Even precisely limited brain regions contain distinct cell populations, which essentially are astrocytes and neurons. These cells present with strikingly different energetic metabolism and thus may develop different responses to the absence of Parkin [53]. Using Seahorse technology to evaluate the respiration of cultured cells in multi-wells plates, we addressed this question by comparing *PARK2^−/−^* and wild type pure primary embryonic neurons or neonatal astrocytes from striatum or cortex (Figure 3). In striatal neurons, the maximal respiration rate and spare respiratory capacity were significantly reduced in the absence of Parkin. In contrast they were similar in *PARK2* ^−/−^ and wild type cortical neurons. Astrocytes strikingly differed from neurons with respect to their uncoupled maximal respiration, which was equal or often lower than their initial respiration. As a result, their spare respiratory capacity appeared null or negative. This observation contrasted with previously reported maximal respiration rates in astrocytes that were at least two-fold higher than basal respiration using Seahorse protocols without the step of oligomycin inhibition [54], [55]. We thus considered that our protocol wasnot suitable for reliable evaluation of maximal respiration and only analyzed initial respiration of astrocytes, which appeared mildly reduced in *PARK2^−/−^* striatum and normal in cortex.

At the end of these diverse analyses of respiration it could be concluded that the absence of parkin was associated with a mild but significant striatal respiratory defect that did not appear to worsen with age and was even detectable in embryonic neurons.

### Mitochondrial inner membrane potential of PARK2^−/−^ mice was normal but showed greater sensitivity to uncoupling with ageing in the striatum

**Figure 4:**
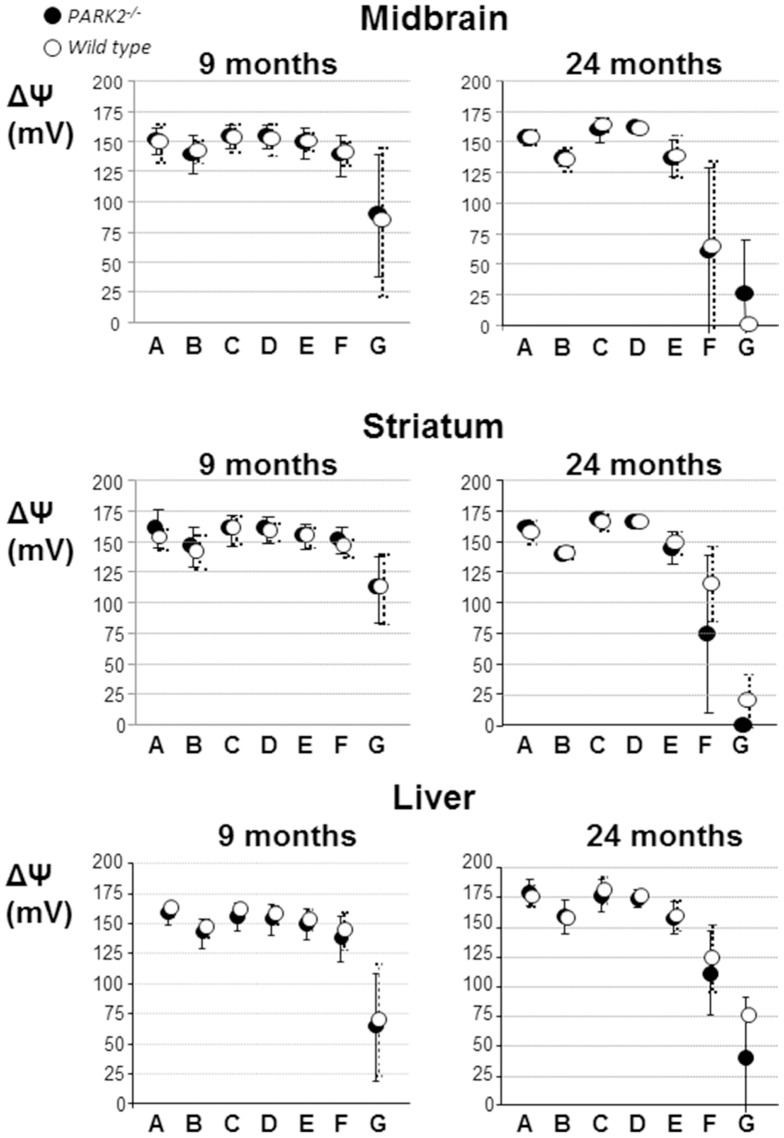
The inner mitochondrial membrane potential is normal in midbrain and striatum of *PARK2* ^−/−^ mice. Dark circles = *PARK2^−/−^*, white circles = wild type; ΔΨm = inner mitochondrial membrane potential expressed as mV after transformation of the fluorescent rhodamine 123 signal using the Nernst equation, as explained in the methods section; A = ΔΨm in the presence of glutamate+malate; B = A+1 mM ADP; C = B+1 µM oligomycin; D = C+1 µM cccp; E = C+2 µM cccp; F = C+4 µM cccp;G = C+8 µM cccp. The data were obtained in parallel to the analysis of respiration; two animals, one per genotype, were analyzed the same day; the data obtained from seven 9-month-old mice and six 24-month-old ones of each genotype, they are expressed as mean and SD.

Low inner membrane potential (Δψm) is one of the expected consequences of a respiration defect. Furthermore it has been proposed to be an important signal for Parkin translocation to mitochondria and mitophagy [56]. To determine whether the absence of Parkin translates *in vivo* into the presence of depolarized mitochondria, we analyzed the Δψm in midbrain, striatum and liver of *PARK2^−/−^* and wild type mice, at 9 and 24 months of age (Figure 4). The reliability of the method was demonstrated in the three tissues by the significant decrease in Δψm upon ADP stimulation (condition B *versus* A, p<0.001 with Mann and Whitney test), its increase upon inhibition of the ATP synthase activity by oligomycin (condition C *versus* B, p<0.001), and decrease with addition of uncoupler above 1 µM (condition C *versus* E, F or G, p<0.001). The mean Δψm did not significantly differ between *PARK2^−/−^* and wild type mice in any of the conditions and tissues examined, excluding excessive accumulation of partially depolarized mitochondria in the absence of Parkin.

In both *PARK2^−/−^* and wild type brain ageing had a clear impact on the Δψm response to increasing doses of the uncoupler cccp with a significant drop of the mean Δψm under 8 µM cccp treatment from 9 to 24 months of age in the striatum and midbrain (p = 0.022 and 0.035 in midbrain and p = 0.001 and <0.001 in striatum of *PARK2^−/−^* and wild type mice respectively using Mann and Whitney test) (Figure 4). The mean Δψm under the lower dose of 4 µM cccp was only significantly lower with age in striatum (p = 0.014 and p = 0.020 in striatum of *PARK2^−/−^* and wild type mice respectively and p = 0.181 and 0.138 in midbrain). The drop of Δψm between condition C (highest value under oligomycin) and condition F or G (uncoupled by either 4 or 8 µM cccp) was then calculated for each mouse. In striatum and under 8 µM cccp only, that drop was significantly higher in *PARK2^−/−^* than in wild type mice (p = 0.041 with Mann and Whitney test).

In liver, despite a similar trend, the changes with age in the mean response of Δψm to uncoupling did not reach significance in wild type or *PARK2^−/−^* mice.

In conclusion the absence of Parkin did not significantly alter the mean Δψm in midbrain, striatum and liver of *PARK2^−/−^* mice but it was associated in striatum with a greater impact of ageing on the Δψm response to uncoupling.

### The respiratory defect in PARK2^−/−^ mice was not due to defective complex I activity, altered mitochondrial content or overt oxidative stress

Complex I activity has previously been reported to be lowered in cells from PD patients with *PARK2* mutations [25], [29], [30] and in a Parkin-depleted zebrafish model [57]. Such a defect may underlie the reduction in striatal tissue respiration and the increased sensitivity of Δψm to uncoupling in *PARK2* ^−/−^ mice. To address this possibility, we used spectrophotometric methods recently standardized for the diagnosis of human mitochondrial diseases [47] to measure the respiratory chain activities in striatal post-nuclear supernatants and crude mitochondrial pellets of cortex and midbrain from one-year-old *PARK2^−/−^* and wild type mice. All the respiratory chain activities examined were similar in *PARK2^−/−^* and wild type mice (Table 1).

**Table 1:** Activities of mitochondrial respiratory chain complexes in 12-month-old *PARK2^−/−^* and wild type mice.

	Striatum		Midbrain		Cortex	
	*PARK2^−/−^*	Wild type	*PARK2^−/−^*	Wild type	*PARK2^−/−^*	Wild type
**Complex I**	91±26	93±20	133±31	138±38	121±27	111±20
**Complex II**	76±24	86±21	248±28	231±64	190±45	183±38
**Combined II+III**	118±42	97±30	304±81	326±86	232±82	283±73
**Complex III**	78±47	60±19	372±121	376±71	696±411	583±154
**Complex IV**	398±78	386±84	1585±598	1875±794	959±221	1112±267
**Complex V**	NA	NA	194±139	171±59	170±94	185±87
**Citrate synthase**	455±33	458±45	194±146	171±162	170±96	185±93

Striatum = post-nuclear supernatants from striatum, Midbrain = crude mitochondrial pellet from ventral midbrain, Cortex = crude mitochondrial pellet from occipital cortex; complex I = rotenone sensitive NADH ubiquinone oxidoreductase, complex II = succinate ubiquinone oxidoreductase, combined II+III = succinate cytochrome c oxidoreductase, complex III = antimycin sensitive ubiquinol cytochrome c oxidoreductase, complex IV = cytochrome c oxidase, complex V = F_1_F_0_ ATPsynthase; complex V assay requires mitochondrial preparation and was therefore not performed on striatal post-nuclear supernatants; data are expressed as nanomoles/minute and mg proteins and as means ± SD; each series of values represents 7 independent samples from each genotype.

Citrate synthase, a citric acid cycle activity often used to evaluate mitochondrial content [58], was also normal in *PARK2^−/−^*. In addition, cell mitochondrial DNA contents were similar in the striatum of *PARK2^−/−^* and wild type mice, confirming that Parkin deficiency does not have an impact on mitochondrial content (1686±119 copies/cell in *PARK2^−/−^* mice *versus* 1791±94 in wild type mice).

**Figure 5:**
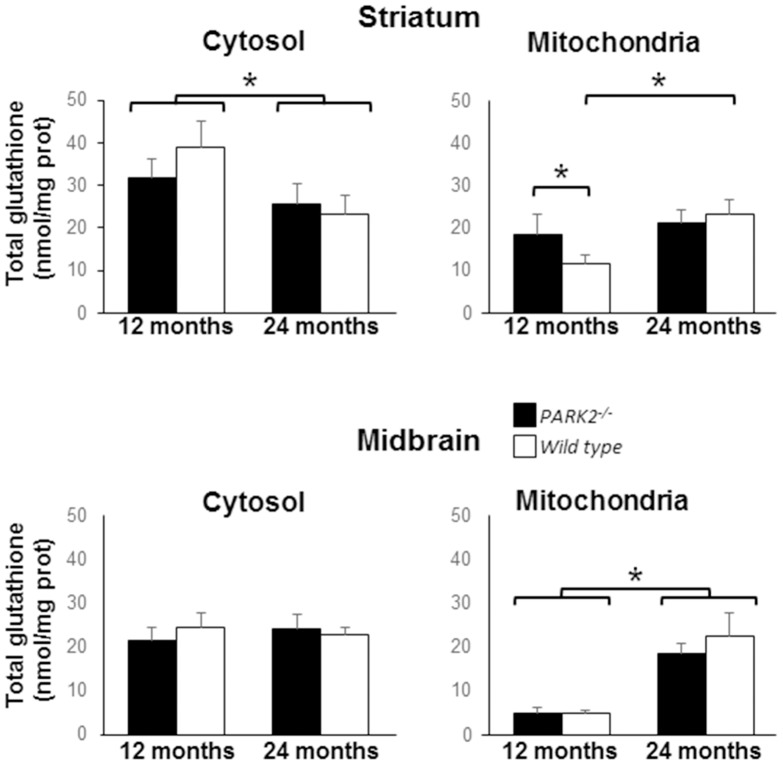
Increase of the mitochondrial glutathione content in striatum of 12-month-old *PARK2* ^−/−^ mice. Total glutathione (GSH+GS-SG) levels were determined in cytosols and isolated mitochondria from ventral midbrain and striatum of 12- and 24-month-old *PARK2^−/−^* (black bars) and wild-type mice (white bars), data are expressed as mean and SD of five independent measurements for each age and each genotype. * = p<0.05 using multiple comparison with Holm-Sidak test. Significant differences with age, without influence of the genotype, were observed for midbrain mitochondrial and striatal cytosolic glutathione content. Significant interaction between age and genotype was present for striatal mitochondrial glutathione content, which significantly increased with age only in wild type mice and was at 12 months of age significantly higher in *PARK2^−/−^* than in wild type mice.

Post-translational modifications regulating the activity of mitochondrial proteins involved in the import, catabolism or oxidative phosphorylation of respiratory substrates could also underlie the respiration defect observed in *PARK2* ^−/−^ mice. Previous studies have reported alterations in proteins involved in the defense against oxidative stress in these mice [35], [36]. We therefore searched for evidence of oxidative stress in midbrain and striatum of *PARK2* ^−/−^ mice. Ageing however being known to have significant influence on oxidative stress, we analyzed the combined influence of age and genotype on the parameters of oxidative stress using multiple comparison by the Holm-Sidak test. Proteasome activity significantly increased with age in striatum (p<0.001) but not midbrain, without influence of the genotype (Table 2). Similarly the midbrain mitochondrial glutathione content increased with age (p<0.001 when comparing 12-month-old to 24 month-old mice) without influence of the genotype). In contrast, significant interaction between age and genotype was observed for the striatal mitochondrial glutathione content (p = 0.015). Ageing led to a significant increase in striatal mitochondrial glutathione in wild type mice (p<0.001 when comparing 12-month-old to 24 month-old wild type mice) but had no effect in *PARK2^−/−^* mice. At 12 months of age the striatal mitochondrial glutathione levels were significantly higher in *PARK2^−/−^* mice than in wild type mice (p<0.011 when comparing 12-month-old wild type to 12-month-old *PARK2^−/−^* mice with Mann and Whitney test). Cytosolic glutathione levels did not change with age in midbrain. In striatum cytosolic glutathione levels significantly decreased with age (p<0.001 when comparing 12-month-old to 24 month-old mice without influence of the genotype, Figure 5); interaction between age and genotype just failed to reach significance (p = 0.056).

**Table 2:** Proteasome activities were similar in *PARK2^−/−^* and wild type mice.

Tissue	Midbrain		Striatum	
Age	12 months	24 months	12 months	24 months
***PARK2^−/−^***	116±89	84±12	43±8	77±110
**wild type**	94±37	80±13	43±4	63±7

Chymotrypsin-like activity of the proteasome. Results are expressed as nmoles of substrate cleaved/minute and milligram of protein and as mean±standard deviation of n = 5 measurements.

**Figure 6:**
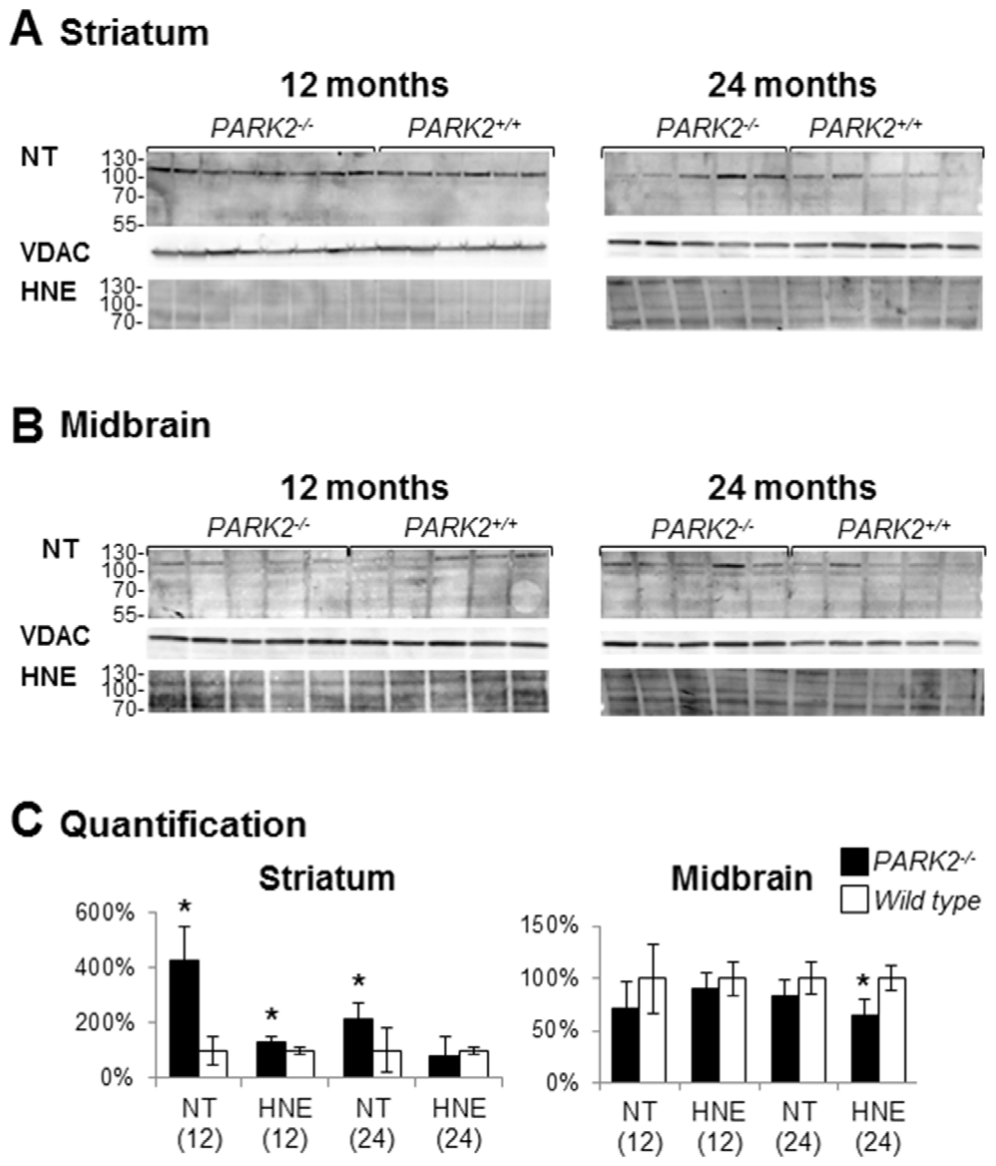
Presence of oxidative adducts in striatal crude mitochondrial pellets from *PARK2* ^−/−^ mice. Western blot analysis of proteins from crude mitochondrial pellets from striatum (A) and midbrain (B) of *PARK2* ^−/−^ and wild-type mice, at 12 and 24 months of age; loading control was the outer membrane protein Voltage-Dependent Anion Channel (VDAC); (C) densitometric analysis of oxidative adducts was performed in the length of the membrane shown in A and B, that signal was then normalized to VDAC signal and quantification was expressed as means and SD and as % of the mean of wild-type samples on the membrane shown in A and B; * = p<0.05 using Mann and Whitney test.

In parallel nitrotyrosine (NT) and 4-hydroxynonenal (HNE), two different protein oxidative adducts, were searched for in crude mitochondrial pellets using western blot analysis. At 12 months of age there was a significant increase in both adducts in the striatum of *PARK2^−/−^* compared to wild type mice (p<0.001 and p = 0.016 for NT and HNE respectively with Mann and Whitney) (Figure 6). At 24 months of age, *PARK2^−/−^* striatum showed increase in NT only (p = 0.028 compared with wild type mice) whereas wild-type mice showed an increase of HNE in midbrain (p = 0.003).

**Figure 7:**
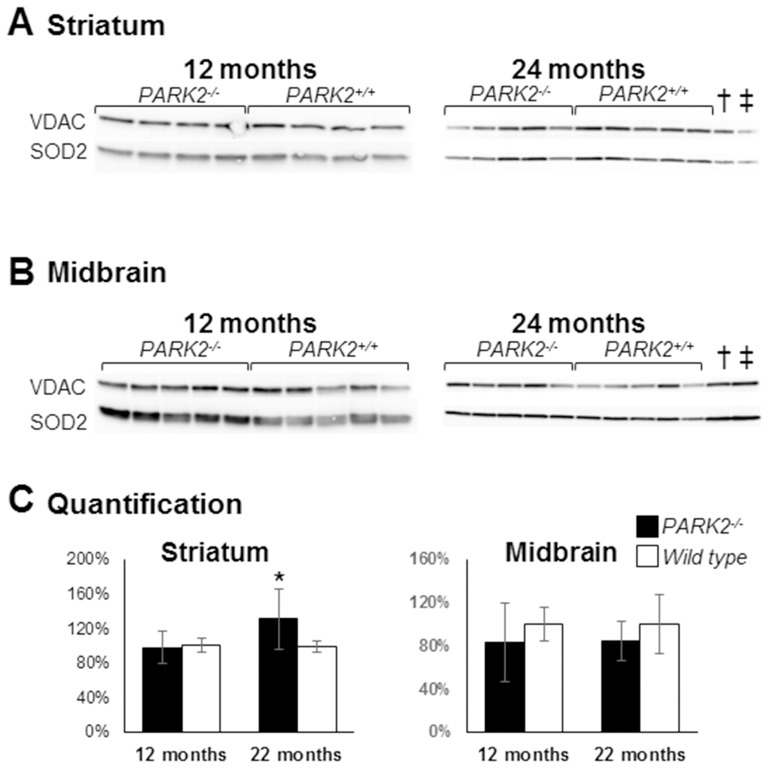
Increased mitochondrial Mn superoxide dismutase (SOD2) in the striatum of *PARK2* ^−/−^ mice. Western blot analysis of proteins from crude mitochondrial pellets from striatum (A) and midbrain (B) of *PARK2* ^−/−^ and wild-type mice, at 12 and 24 months of age; loading control was the outer membrane protein Voltage-Dependent Anion Channel (VDAC); (C) densitometric analysis of the SOD2 signal shown in A and B was normalized to VDAC signal and quantification was expressed as means and SD and as % of the mean of wild-type samples on the membrane shown in A and B; * = p<0.05 using Mann and Whitney test; † and ‡: samples from 12-month-old *PARK2^−/−^* and wild type mice respectively showing that the SOD2 steady-state level does not significantly change with age.

The steady-state of the mitochondrial superoxide dismutase 2 (SOD2), normally increased in the presence of mitochondrial oxidative stress [59], was normal at 12 months of age and slightly increased at 24 months of age in *PARK2^−/−^* mice compared to wild type mice (p = 0.016 using Mann and Whitney test) (Figure 7).

Altogether, our results show an increase in oxidative stress in the striatum mitochondria of *PARK2^−/−^* mice (increased glutathione levels, increased oxidative adducts and, with age, increased SOD2 expression). That increase is however moderate (normal SOD2 expression at 12 months of age, normal proteasome activity).

## Discussion

Recent work, based on the analysis of cell models overproducing Parkin and subjected to pharmacological treatments leading to immediate and global depolarization of the mitochondrial network, attributed to Parkin a central role in the clearance of dysfunctional mitochondria [12]. These studies predict that loss of function of the PINK1/Parkin pathway should lead to progressive mitochondrial impairment, possibly representing a primary mechanism of neurodegeneration in autosomal recessive PD. However, whether Parkin-dependent mitochondrial quality control plays indeed a role *in vivo* under physiological conditions and during aging, remains debated.

We addressed this issue by revisiting mitochondrial functions in *PARK2^−/−^* mice using recently developed, robust and sensitive technologies. The simultaneous analysis of minimal amounts of post-nuclear supernatants from different tissues with high resolution respirometry revealed a reliable respiratory defect specifically in the striatum of *PARK2^−/−^* mice, which was not exacerbated by ageing. The presence of this defect was confirmed in primary embryonic striatal neurons and was not found in cortical neurons from *PARK2^−/−^* mice using the Seahorse technology, supporting its region-specificity and early appearance in life.

Contrary to previous observations in cell and zebrafish models [25], [29], [57], the constitutive deletion of *PARK2* in mice did not result in significant changes in mitochondrial membrane potential, overt oxidative stress markers or defective activity of mitochondrial complex I or other respiratory chain complexes despite the presence of significant respiratory defect. With ageing however the mitochondrial potential in the striatum showed increased sensitivity to uncoupling, with a greater impact in *PARK2^−/−^* than in wild type mice, indicating less efficient compensation for an induced proton leak. As this defect cannot be ascribed to an age-dependent decline of respiratory capacity, it may be secondary to an age-dependent alteration of the inner membrane composition or defect in OXPHOS substrate transport or oxidation, all of which could add a burden to the preexisting respiration defect in *PARK2^−/−^* mice.

The relatively mild modifications of mitochondrial function in *PARK2^−/−^* mice suggest that Parkin does not play a predominant role in base-line mitochondrial quality control *in vivo*. Its constitutive depletion may be compensated for by parallel, redundant or alternative mitochondrial quality control mechanisms, already proposed to intervene following PINK1 depletion [60]. This possibility is supported by the recent observation in the heart of *PARK2^−/−^* mice showing absence of a functional defect of mitochondria in the myocardium under basal conditions but defective clearing of damaged mitochondria following myocardiac infarction [61] and accumulation of mitochondria with abnormal ultrastructure in myocytes during aging [62]. Therefore, as in cardiac tissue, the mitochondrial quality control function of the PINK1/Parkin pathway may become essential in the central nervous system under stress conditions, for example following accumulation of oxidative stress during ageing [63]. Consistent with this possibility, mitochondrial stress caused by the conditional expression of mitochondrial unfolded ornithine transcarbamylase has been recently shown to lead to dopaminergic neurodegeneration and a parkinsonian phenotype in *PINK1−/−* mice [64].

Contrary to humans, the short life-time of rodents may not allow full unmasking of a phenotype following disruption of the PINK1/Parkin pathway, explaining the moderate mitochondrial defect and lack of overt neurodegeneration in *PARK2−/−* mice. Signs of oxidative damage were mild in aged *PARK2−/−* mice. Compensatory mechanisms such as the increase in glutathione levels, specifically in the mitochondrial fraction, may enhance the cellular antioxidant capacity and counteract oxidative stress in these mice. The relative content of reduced and oxidized glutathione should be determined in this fraction to properly evaluate the efficiency of this mechanism. Of note, we previously reported a selective increase in reduced glutathione content in total extracts from striatum and primary mesencephalic neurons of *PARK2−/−* mice [39]. The increase of SOD2, often used as a marker of oxidative stress, is also a protective mechanism efficiently neutralizing superoxide anions.

In conclusion, our work revealed an early respiration defect in *PARK2−/−* mice that may reflect a disease-relevant preclinical modification in autosomal recessive PD. Future studies are required to identify the molecular mechanisms underlying this modification and determine their relation to the mitochondrial quality control activity of the PINK1/Parkin pathway. To fully appreciate the role of PINK1/Parkin-dependent mitochondrial quality control in the physiopathology of autosomal recessive PD, it will also be essential to determine whether stress conditions exacerbate mitochondrial dysfunction and unmask a neurodegenerative phenotype in the brains of *PARK2^−/−^* mice.

## Supporting Information

Figure S1 **Scheme of the different brain fractionation methods used to assess mitochondrial functions.**
(TIF)

Figure S2 **Respiratory control ratios (RCR) in 24-month-old mice.** Respiratory control ratios were calculated as state 3/state 4 respiration rate on glutamate+malate as substrates; results are shown as mean and SD; numbers between brackets indicate the number of independent measurements in individual animals of each genotype (**A**) crude mitochondrial pellets from cortex (n = 8), crude mitochondrial pellets from striatum (n = 6) and purified mitochondrial pelllets from whole brain (n = 2); (**B**) post-nuclear supernatants from striatum (n = 7), midbrain (n = 8) and liver (n = 8).
(TIF)

## References

[1] Schapira AH, Cooper JM, Dexter D, Jenner P, Clark JB, et al (1989) Mitochondrial complex I deficiency in Parkinson's disease. Lancet 1: 1269. 2566813 10.1016/s0140-6736(89)92366-0

[2] Mann VM, Cooper JM, Krige D, Daniel SE, Schapira AHV, et al (1992) Brain, skeletal muscle and platelet homogenate mitochondrial function in Parkinson's disease. Brain 115: ?. 10.1093/brain/115.2.333 1606472

[3] Hauser DN, Hastings TG (2013) Mitochondrial dysfunction and oxidative stress in Parkinson's disease and monogenic parkinsonism. Neurobiol Dis 51: 35–42. 23064436 10.1016/j.nbd.2012.10.011 PMC3565564

[4] Cardoso SM (2011) The mitochondrial cascade hypothesis for Parkinson's disease. Curr Pharm Des 17: 3390–3397. 21902669 10.2174/138161211798072508

[5] Corti O, Lesage S, Brice A (2011) What genetics tells us about the causes and mechanisms of Parkinson's disease. Physiol Rev 91: 1161–1218. 22013209 10.1152/physrev.00022.2010

[6] Darios F, Corti O, Lucking CB, Hampe C, Muriel MP, et al (2003) Parkin prevents mitochondrial swelling and cytochrome c release in mitochondria-dependent cell death. Hum Mol Genet 12: 517–526. 12588799 10.1093/hmg/ddg044

[7] Canet-Aviles RM, Wilson MA, Miller DW, Ahmad R, McLendon C, et al (2004) The Parkinson's disease protein DJ-1 is neuroprotective due to cysteine-sulfinic acid-driven mitochondrial localization. Proc Natl Acad Sci U S A 101: 9103–9108. 15181200 10.1073/pnas.0402959101 PMC428480

[8] Valente EM, Abou-Sleiman PM, Caputo V, Muqit MM, Harvey K, et al (2004) Hereditary early-onset Parkinson's disease caused by mutations in PINK1. Science 304: 1158–1160. 15087508 10.1126/science.1096284

[9] Corti O, Brice A (2013) Mitochondrial quality control turns out to be the principal suspect in parkin and PINK1-related autosomal recessive Parkinson's disease. Curr Opin Neurobiol 23: 100–108. 23206589 10.1016/j.conb.2012.11.002

[10] Narendra D, Tanaka A, Suen DF, Youle RJ (2008) Parkin is recruited selectively to impaired mitochondria and promotes their autophagy. J Cell Biol 183: 795–803. 19029340 10.1083/jcb.200809125 PMC2592826

[11] Narendra DP, Jin SM, Tanaka A, Suen DF, Gautier CA, et al (2010) PINK1 is selectively stabilized on impaired mitochondria to activate Parkin. PLoS Biol 8: e1000298. 20126261 10.1371/journal.pbio.1000298 PMC2811155

[12] Narendra D, Walker JE, Youle R (2012) Mitochondrial quality control mediated by PINK1 and Parkin: links to parkinsonism. Cold Spring Harb Perspect Biol 4. 10.1101/cshperspect.a011338 PMC3536340 23125018

[13] Vives-Bauza C, Zhou C, Huang Y, Cui M, de Vries RL, et al (2010) PINK1-dependent recruitment of Parkin to mitochondria in mitophagy. Proc Natl Acad Sci U S A 107: 378–383. 19966284 10.1073/pnas.0911187107 PMC2806779

[14] Geisler S, Holmstrom KM, Treis A, Skujat D, Weber SS, et al (2010) The PINK1/Parkin-mediated mitophagy is compromised by PD-associated mutations. Autophagy 6: 871–878. 20798600 10.4161/auto.6.7.13286

[15] Matsuda N, Sato S, Shiba K, Okatsu K, Saisho K, et al (2010) PINK1 stabilized by mitochondrial depolarization recruits Parkin to damaged mitochondria and activates latent Parkin for mitophagy. J Cell Biol 189: 211–221. 20404107 10.1083/jcb.200910140 PMC2856912

[16] Yang Y, Gehrke S, Imai Y, Huang Z, Ouyang Y, et al (2006) Mitochondrial pathology and muscle and dopaminergic neuron degeneration caused by inactivation of Drosophila Pink1 is rescued by Parkin. Proc Natl Acad Sci U S A 103: 10793–10798. 16818890 10.1073/pnas.0602493103 PMC1502310

[17] Poole AC, Thomas RE, Andrews LA, McBride HM, Whitworth AJ, et al (2008) The PINK1/Parkin pathway regulates mitochondrial morphology. Proc Natl Acad Sci U S A 105: 1638–1643. 18230723 10.1073/pnas.0709336105 PMC2234197

[18] Deng H, Dodson MW, Huang H, Guo M (2008) The Parkinson's disease genes pink1 and parkin promote mitochondrial fission and/or inhibit fusion in Drosophila. Proc Natl Acad Sci U S A 105: 14503–14508. 18799731 10.1073/pnas.0803998105 PMC2567186

[19] Wang X, Winter D, Ashrafi G, Schlehe J, Wong YL, et al (2011) PINK1 and Parkin target Miro for phosphorylation and degradation to arrest mitochondrial motility. Cell 147: 893–906. 22078885 10.1016/j.cell.2011.10.018 PMC3261796

[20] Liu S, Sawada T, Lee S, Yu W, Silverio G, et al (2012) Parkinson's disease-associated kinase PINK1 regulates Miro protein level and axonal transport of mitochondria. PLoS Genet 8: e1002537. 22396657 10.1371/journal.pgen.1002537 PMC3291531

[21] Shin JH, Ko HS, Kang H, Lee Y, Lee YI, et al (2011) PARIS (ZNF746) repression of PGC-1alpha contributes to neurodegeneration in Parkinson's disease. Cell 144: 689–702. 21376232 10.1016/j.cell.2011.02.010 PMC3063894

[22] Pacelli C, De Rasmo D, Signorile A, Grattagliano I, di Tullio G, et al (2011) Mitochondrial defect and PGC-1alpha dysfunction in parkin-associated familial Parkinson's disease. Biochim Biophys Acta 1812: 1041–1053. 21215313 10.1016/j.bbadis.2010.12.022

[23] Exner N, Treske B, Paquet D, Holmstrom K, Schiesling C, et al (2007) Loss-of-function of human PINK1 results in mitochondrial pathology and can be rescued by parkin. J Neurosci 27: 12413–12418. 17989306 10.1523/JNEUROSCI.0719-07.2007 PMC6673250

[24] Gandhi S, Wood-Kaczmar A, Yao Z, Plun-Favreau H, Deas E, et al (2009) PINK1-associated Parkinson's disease is caused by neuronal vulnerability to calcium-induced cell death. Mol Cell 33: 627–638. 19285945 10.1016/j.molcel.2009.02.013 PMC2724101

[25] Mortiboys H, Thomas KJ, Koopman WJ, Klaffke S, Abou-Sleiman P, et al (2008) Mitochondrial function and morphology are impaired in parkin-mutant fibroblasts. Ann Neurol 64: 555–565. 19067348 10.1002/ana.21492 PMC2613566

[26] Morais VA, Verstreken P, Roethig A, Smet J, Snellinx A, et al (2009) Parkinson's disease mutations in PINK1 result in decreased Complex I activity and deficient synaptic function. EMBO Mol Med 1: 99–111. 20049710 10.1002/emmm.200900006 PMC3378121

[27] Zhang C, Lin M, Wu R, Wang X, Yang B, et al (2011) Parkin, a p53 target gene, mediates the role of p53 in glucose metabolism and the Warburg effect. Proc Natl Acad Sci U S A 108: 16259–16264. 21930938 10.1073/pnas.1113884108 PMC3182683

[28] Grunewald A, Gegg ME, Taanman JW, King RH, Kock N, et al (2009) Differential effects of PINK1 nonsense and missense mutations on mitochondrial function and morphology. Exp Neurol 219: 266–273. 19500570 10.1016/j.expneurol.2009.05.027

[29] Grunewald A, Voges L, Rakovic A, Kasten M, Vandebona H, et al (2010) Mutant Parkin impairs mitochondrial function and morphology in human fibroblasts. PLoS One 5: e12962. 20885945 10.1371/journal.pone.0012962 PMC2946349

[30] Muftuoglu M, Elibol B, Dalmizrak O, Ercan A, Kulaksiz G, et al (2004) Mitochondrial complex I and IV activities in leukocytes from patients with parkin mutations. Mov Disord 19: 544–548. 15133818 10.1002/mds.10695

[31] Sterky FH, Lee S, Wibom R, Olson L, Larsson NG (2011) Impaired mitochondrial transport and Parkin-independent degeneration of respiratory chain-deficient dopamine neurons in vivo. Proc Natl Acad Sci U S A 108: 12937–12942. 21768369 10.1073/pnas.1103295108 PMC3150929

[32] Van Laar VS, Arnold B, Cassady SJ, Chu CT, Burton EA, et al (2011) Bioenergetics of neurons inhibit the translocation response of Parkin following rapid mitochondrial depolarization. Hum Mol Genet 20: 927–940. 21147754 10.1093/hmg/ddq531 PMC3033183

[33] Gautier CA, Kitada T, Shen J (2008) Loss of PINK1 causes mitochondrial functional defects and increased sensitivity to oxidative stress. Proc Natl Acad Sci U S A 105: 11364–11369. 18687901 10.1073/pnas.0802076105 PMC2516271

[34] Gispert S, Ricciardi F, Kurz A, Azizov M, Hoepken HH, et al (2009) Parkinson phenotype in aged PINK1-deficient mice is accompanied by progressive mitochondrial dysfunction in absence of neurodegeneration. PLoS One 4: e5777. 19492057 10.1371/journal.pone.0005777 PMC2686165

[35] Palacino JJ, Sagi D, Goldberg MS, Krauss S, Motz C, et al (2004) Mitochondrial dysfunction and oxidative damage in parkin-deficient mice. J Biol Chem 279: 18614–18622. 14985362 10.1074/jbc.M401135200

[36] Periquet M, Corti O, Jacquier S, Brice A (2005) Proteomic analysis of parkin knockout mice: alterations in energy metabolism, protein handling and synaptic function. J Neurochem 95: 1259–1276. 16150055 10.1111/j.1471-4159.2005.03442.x

[37] Stichel CC, Zhu XR, Bader V, Linnartz B, Schmidt S, et al (2007) Mono- and double-mutant mouse models of Parkinson's disease display severe mitochondrial damage. Hum Mol Genet 16: 2377–2393. 17412759 10.1093/hmg/ddm083

[38] Goldberg MS, Fleming SM, Palacino JJ, Cepeda C, Lam HA, et al (2003) Parkin-deficient mice exhibit nigrostriatal deficits but not loss of dopaminergic neurons. J Biol Chem 278: 43628–43635. 12930822 10.1074/jbc.M308947200

[39] Itier JM, Ibanez P, Mena MA, Abbas N, Cohen-Salmon C, et al (2003) Parkin gene inactivation alters behaviour and dopamine neurotransmission in the mouse. Hum Mol Genet 12: 2277–2291. 12915482 10.1093/hmg/ddg239

[40] Von Coelln R, Thomas B, Savitt JM, Lim KL, Sasaki M, et al (2004) Loss of locus coeruleus neurons and reduced startle in parkin null mice. Proc Natl Acad Sci U S A 101: 10744–10749. 15249681 10.1073/pnas.0401297101 PMC490005

[41] Perez FA, Palmiter RD (2005) Parkin-deficient mice are not a robust model of parkinsonism. Proc Natl Acad Sci U S A 102: 2174–2179. 15684050 10.1073/pnas.0409598102 PMC548311

[42] Fournier M, Vitte J, Garrigue J, Langui D, Dullin JP, et al (2009) Parkin deficiency delays motor decline and disease manifestation in a mouse model of synucleinopathy. PLoS One 4: e6629. 19680561 10.1371/journal.pone.0006629 PMC2722082

[43] Chinopoulos C, Zhang SF, Thomas B, Ten V, Starkov AA (2011) Isolation and functional assessment of mitochondria from small amounts of mouse brain tissue. Methods Mol Biol 793: 311–324. 21913109 10.1007/978-1-61779-328-8_20 PMC3627350

[44] Friedman WJ, Ibanez CF, Hallbook F, Persson H, Cain LD, et al (1993) Differential actions of neurotrophins in the locus coeruleus and basal forebrain. Exp Neurol 119: 72–78. 8432352 10.1006/exnr.1993.1007

[45] Morganti MC, Taylor J, Pesheva P, Schachner M (1990) Oligodendrocyte-derived J1-160/180 extracellular matrix glycoproteins are adhesive or repulsive depending on the partner cell type and time of interaction. Exp Neurol 109: 98–110. 2192910 10.1016/s0014-4886(05)80012-3

[46] Wu M, Neilson A, Swift AL, Moran R, Tamagnine J, et al (2007) Multiparameter metabolic analysis reveals a close link between attenuated mitochondrial bioenergetic function and enhanced glycolysis dependency in human tumor cells. Am J Physiol Cell Physiol 292: C125–136. 16971499 10.1152/ajpcell.00247.2006

[47] Medja F, Allouche S, Frachon P, Jardel C, Malgat M, et al (2009) Development and implementation of standardized respiratory chain spectrophotometric assays for clinical diagnosis. Mitochondrion 9: 331–339. 19439198 10.1016/j.mito.2009.05.001

[48] Schagger H, Pfeiffer K (2001) The ratio of oxidative phosphorylation complexes I–V in bovine heart mitochondria and the composition of respiratory chain supercomplexes. J Biol Chem 276: 37861–37867. 11483615 10.1074/jbc.M106474200

[49] Couplan E, Gelly C, Goubern M, Fleury C, Quesson B, et al (2002) High level of uncoupling protein 1 expression in muscle of transgenic mice selectively affects muscles at rest and decreases their IIb fiber content. J Biol Chem 277: 43079–43088. 12221093 10.1074/jbc.M206726200

[50] Viengchareun S, Caron M, Auclair M, Kim MJ, Frachon P, et al (2007) Mitochondrial toxicity of indinavir, stavudine and zidovudine involves multiple cellular targets in white and brown adipocytes. Antivir Ther 12: 919–929. 17926646

[51] Auchere F, Santos R, Planamente S, Lesuisse E, Camadro JM (2008) Glutathione-dependent redox status of frataxin-deficient cells in a yeast model of Friedreich's ataxia. Hum Mol Genet 17: 2790–2802. 18562474 10.1093/hmg/ddn178

[52] Bulteau AL, Dancis A, Gareil M, Montagne JJ, Camadro JM, et al (2007) Oxidative stress and protease dysfunction in the yeast model of Friedreich ataxia. Free Radic Biol Med 42: 1561–1570. 17448903 10.1016/j.freeradbiomed.2007.02.014

[53] Magistretti PJ, Pellerin L (1996) Cellular mechanisms of brain energy metabolism. Relevance to functional brain imaging and to neurodegenerative disorders. Ann N Y Acad Sci 777: 380–387. 8624117 10.1111/j.1749-6632.1996.tb34449.x

[54] Larsen NJ, Ambrosi G, Mullett SJ, Berman SB, Hinkle DA (2011) DJ-1 knock-down impairs astrocyte mitochondrial function. Neuroscience 196: 251–264. 21907265 10.1016/j.neuroscience.2011.08.016 PMC3490195

[55] Gimenez-Cassina A, Martinez-Francois JR, Fisher JK, Szlyk B, Polak K, et al (2012) BAD-dependent regulation of fuel metabolism and K(ATP) channel activity confers resistance to epileptic seizures. Neuron 74: 719–730. 22632729 10.1016/j.neuron.2012.03.032 PMC3361694

[56] Chen H, Chan DC (2009) Mitochondrial dynamics–fusion, fission, movement, and mitophagy–in neurodegenerative diseases. Hum Mol Genet 18: R169–176. 19808793 10.1093/hmg/ddp326 PMC2758711

[57] Flinn L, Mortiboys H, Volkmann K, Koster RW, Ingham PW, et al (2009) Complex I deficiency and dopaminergic neuronal cell loss in parkin-deficient zebrafish (Danio rerio). Brain 132: 1613–1623. 19439422 10.1093/brain/awp108

[58] Kirby DM, Thorburn DR, Turnbull DM, Taylor RW (2007) Biochemical assays of respiratory chain complex activity. Methods Cell Biol 80: 93–119. 17445690 10.1016/S0091-679X(06)80004-X

[59] Macmillan-Crow LA, Cruthirds DL (2001) Invited review: manganese superoxide dismutase in disease. Free Radic Res 34: 325–336. 11328670 10.1080/10715760100300281

[60] Dagda RK, Gusdon AM, Pien I, Strack S, Green S, et al (2011) Mitochondrially localized PKA reverses mitochondrial pathology and dysfunction in a cellular model of Parkinson's disease. Cell Death Differ 18: 1914–1923. 21637291 10.1038/cdd.2011.74 PMC3177020

[61] Kubli DA, Zhang X, Lee Y, Hanna RA, Quinsay MN, et al (2013) Parkin protein deficiency exacerbates cardiac injury and reduces survival following myocardial infarction. J Biol Chem 288: 915–926. 23152496 10.1074/jbc.M112.411363 PMC3543040

[62] Kubli DA, Quinsay MN, Gustafsson AB (2013) Parkin deficiency results in accumulation of abnormal mitochondria in aging myocytes. Commun Integr Biol 6: e24511. 23986804 10.4161/cib.24511 PMC3737749

[63] Harman D (1972) The biologic clock: the mitochondria? J Am Geriatr Soc 20: 145–147. 5016631 10.1111/j.1532-5415.1972.tb00787.x

[64] Moisoi N, Fedele V, Edwards J, Martins LM (2014) Loss of PINK1 enhances neurodegeneration in a mouse model of Parkinson's disease triggered by mitochondrial stress. Neuropharmacology 77: 350–357. 24161480 10.1016/j.neuropharm.2013.10.009 PMC3878764

