# Supplementary figures and images for: Tissue- and Cell-Specific Mitochondrial Defect in Parkin-Deficient Mice
Source: PLoS One. 2014 Jun 24;9(6):e99898. doi: 10.1371/journal.pone.0099898 (PMC4069072; doi:10.1371/journal.pone.0099898)

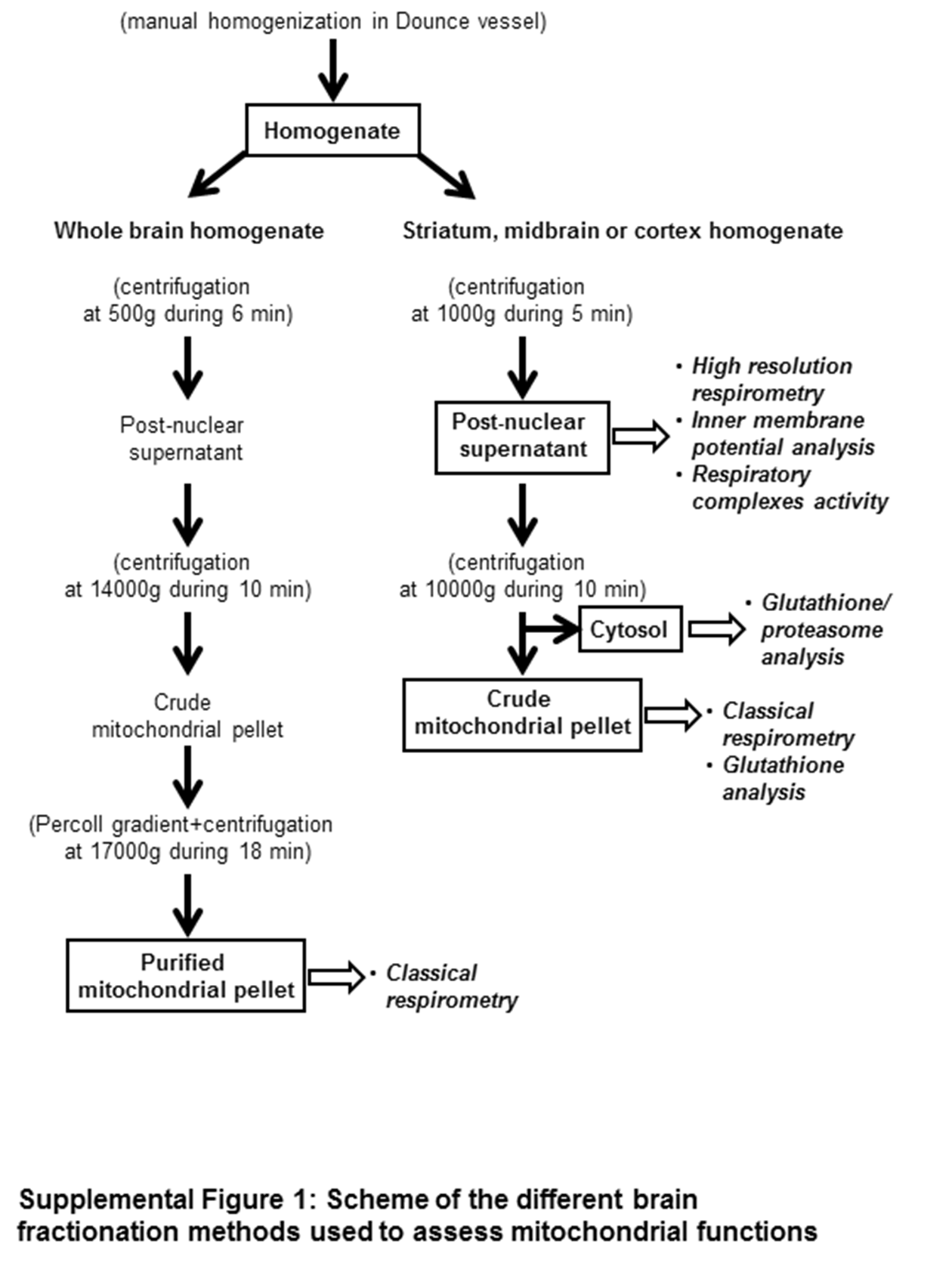

Supplement: Figure S1 — Scheme of the different brain fractionation methods used to assess mitochondrial functions. (TIF) [file pone.0099898.s001.tif]

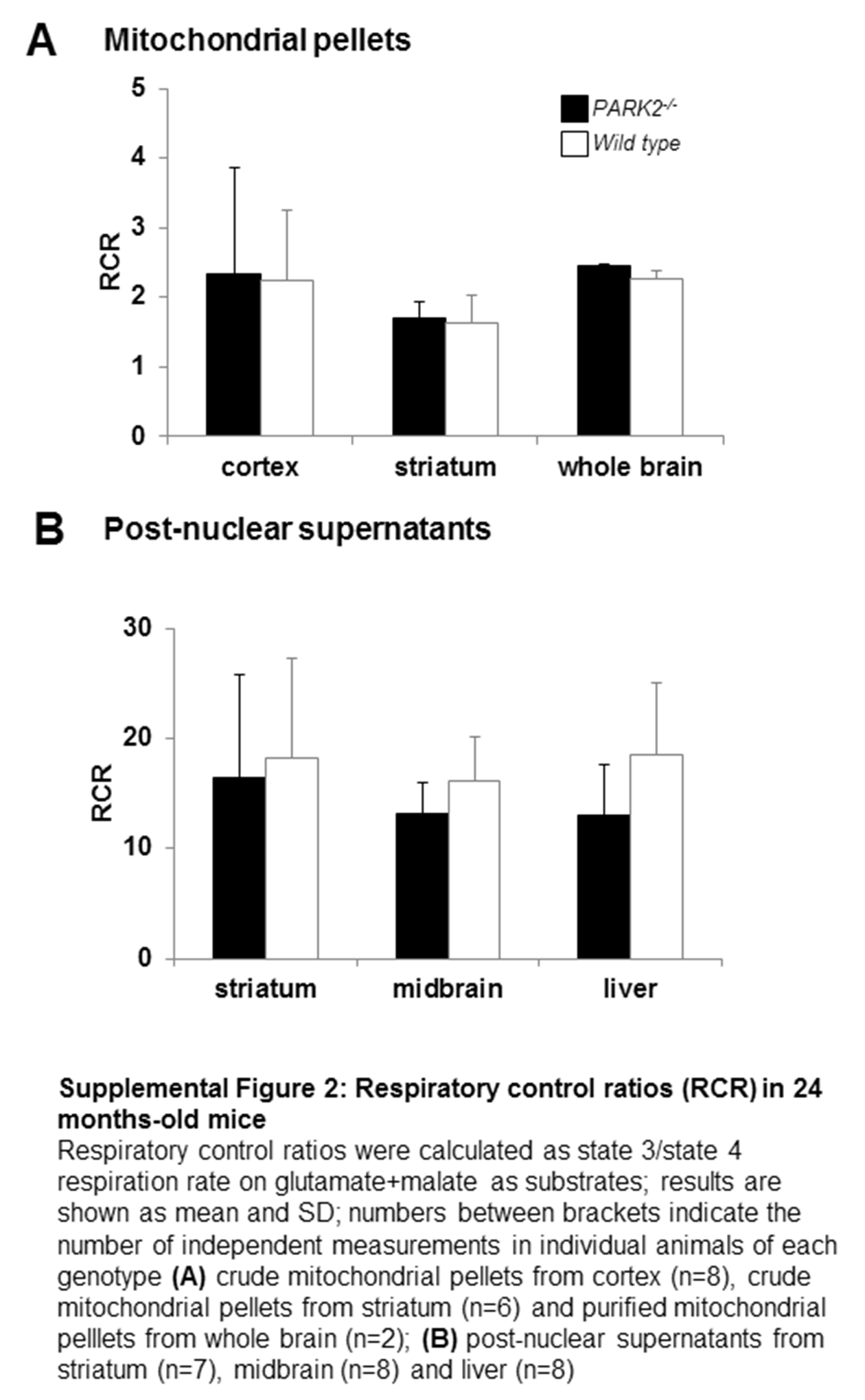

Supplement: Figure S2 — Respiratory control ratios (RCR) in 24-month-old mice. Respiratory control ratios were calculated as state 3/state 4 respiration rate on glutamate+malate as substrates; results are shown as mean and SD; numbers between brackets indicate the number of independent measurements in individual animals of each genotype (A) crude mitochondrial pellets from cortex (n = 8), crude mitochondrial pellets from striatum (n = 6) and purified mitochondrial pelllets from whole brain (n = 2); (B) post-nuclear supernatants from striatum (n = 7), midbrain (n = 8) and liver (n = 8). (TIF) [file pone.0099898.s002.tif]
